# Compound Heterozygous Sickle Cell-Beta Thalassemia Presenting As Chronic Hemolytic Anemia With Microcytosis and Prominent Left Ventricular Trabeculation: A Case Report

**DOI:** 10.7759/cureus.108797

**Published:** 2026-05-13

**Authors:** Vijay Krishnan Radhakrishnan, Aswanaa Kamanuru Govindarajulu, Thilakavathi N Rajendran, Janani Nabirajan, Suganya Balachandran

**Affiliations:** 1 Internal Medicine, Sri Lalithambigai Medical College and Hospital, Chennai, IND

**Keywords:** beta thalassemia, hemoglobinopathy, hemolytic anemia, left ventricular trabeculation, microcytosis, sickle cell disease

## Abstract

Sickle cell-beta thalassemia, a compound heterozygous hemoglobinopathy, can be difficult to diagnose because clinical features and laboratory findings often overlap with other hematological disorders. We report a 26-year-old man from Assam who presented with eight days of progressive jaundice. Further evaluation revealed chronic hemolysis that had gone undiagnosed for years. He also reported one week of abdominal distension and generalized weakness. Clinical examination revealed pallor, icterus, and moderate splenomegaly.

Laboratory evaluation demonstrated anemia (hemoglobin 6.3-7.9 g/dL), microcytosis, elevated lactate dehydrogenase, indirect hyperbilirubinemia, low serum haptoglobin, moderate splenomegaly and reticulocytosis, consistent with ongoing hemolysis. The direct Coombs test was negative, ruling out immune-mediated hemolysis. Infectious causes, including chronic malaria, were excluded, and serological tests for HIV, hepatitis B, and hepatitis C were negative. Blood and urine cultures showed no growth. Glucose-6-phosphate dehydrogenase (G6PD) levels and iron studies were within normal limits, excluding enzymopathy and iron deficiency.

Hemoglobin analysis revealed hemoglobin S (HbS) 73.1%, fetal hemoglobin (HbF) 15%, hemoglobin A2 (HbA2) 6.3%, and reduced hemoglobin A (HbA), consistent with compound heterozygous sickle cell-beta thalassemia. Echocardiography demonstrated prominent left ventricular apical trabeculations, likely representing adaptive cardiac remodeling in response to a chronic high-output state.

This case illustrates how systematic workup can identify compound hemoglobinopathies in patients with atypical presentations. The finding of HbS-β thalassemia in a patient from Assam is epidemiologically significant, as this compound hemoglobinopathy is extremely rare in this region, where HbE variants predominate. The cardiac findings might reflect cardiovascular changes from chronic, untreated anemia.

## Introduction

Hemoglobinopathies are among the most common inherited disorders worldwide, particularly in developing countries [1]. Compound heterozygous states such as sickle cell-beta thalassemia exhibit a wide spectrum of clinical severity depending on beta-globin chain production [2,3].

The clinical presentation often shows overlapping features of hemolysis and ineffective erythropoiesis, making it essential to distinguish this from other causes of hemolytic anemia. A systematic diagnostic approach is therefore required to exclude infectious, immune, and secondary causes.

Sickle cell-beta thalassemia results from compound heterozygosity, where an individual inherits the sickle cell gene (HbS) from one parent and a beta-thalassemia mutation from the other parent. The condition is classified into two major types: sickle cell-β0-thalassemia, characterized by the complete absence of normal beta-globin chain production (HbA = 0%), and sickle cell-β+ thalassemia, where reduced but detectable beta-globin synthesis occurs (HbA typically 5-30%). This genetic distinction is clinically significant, as β0-thalassemia generally presents with more severe manifestations similar to homozygous sickle cell disease (HbSS), while β+ thalassemia often exhibits a milder phenotype with better clinical outcomes.

We present a case of chronic hemolytic anemia in a young adult, in whom sequential evaluation led to the diagnosis of compound heterozygous sickle cell-beta thalassemia, with an additional finding of prominent left ventricular trabeculation representing likely adaptive cardiac remodeling.

## Case presentation

A 26-year-old man from Assam presented with yellowish discoloration of the eyes for eight days, abdominal distension for one week, and generalized weakness. The patient had chronic hemolysis that was never previously diagnosed, and this acute episode brought him to medical attention. The patient's origin from Assam is epidemiologically relevant, as this region shows a distinct hemoglobinopathy pattern with HbE-β thalassemia being far more prevalent than HbS-β thalassemia, making this case particularly unusual for the region [4,5].

He denied fever, bleeding, tattooing, or previous transfusions. The patient reported intermittent jaundice during childhood that had not been evaluated. There was no history or clinical evidence of chronic leg ulcers and bony deformities at presentation. Family history revealed one sibling who was asymptomatic; screening was advised. On examination, the patient was pale and icteric. Abdominal examination revealed moderate splenomegaly. There was no lymphadenopathy or evidence of acute vaso-occlusive crisis.

Investigations

Hematological Findings

Complete blood count revealed hemoglobin of 6.3-7.9 g/dL with a mean corpuscular volume (MCV) of 65-70 fL and elevated red cell distribution width (RDW). Reticulocyte count was 5%. The direct Coombs test was negative. These findings were suggestive of non-immune hemolytic anemia (Table 1).

**Table 1:** Hematological parameters at presentation. RBC: red blood cell; PCV: packed cell volume; MCV: mean corpuscular volume; MCH: mean corpuscular hemoglobin; RDW: red cell distribution width

Parameter	Value	Reference Range	Interpretation
Hemoglobin	6.3-7.9 g/dL (trend)	13-17 g/dL	Severe anemia
RBC count	2.84-3.65 ×10^6^/µL	4.5-5.5 ×10^6^/µL	Reduced
PCV	18.5-25.6%	40-50%	Low
MCV	65-70 fL	80-100 fL	Microcytic
MCH	~22 pg	27-32 pg	Hypochromic
RDW	28.1%	11-14%	Elevated
Reticulocyte count	5%	0.5-2.5%	Increased

Peripheral Smear Findings

**Figure 1:**
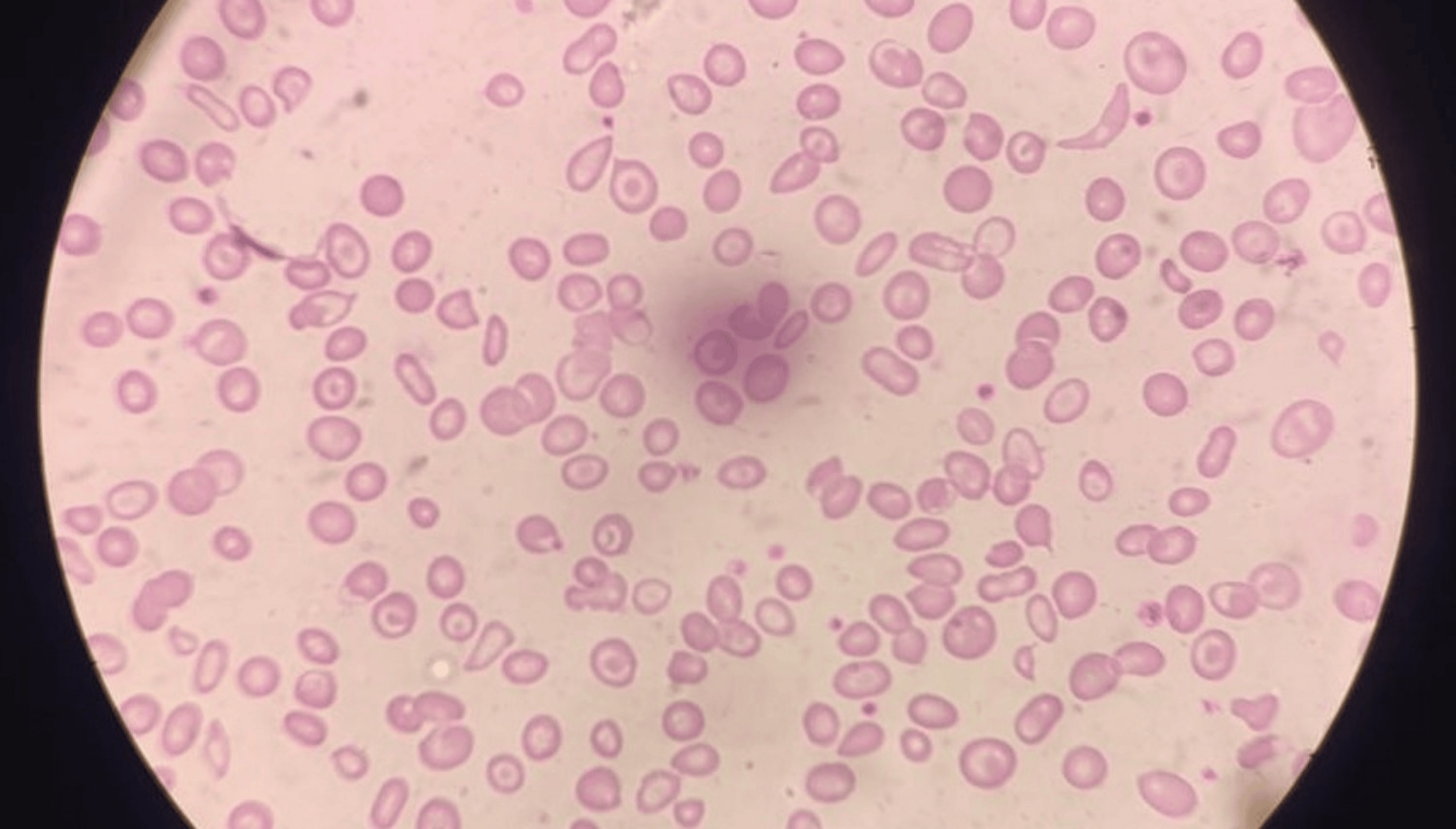
Peripheral blood smear showing microcytic hypochromic red blood cells with marked anisopoikilocytosis, including target cells, teardrop cells, and occasional sickle cells and schistocytes.

Peripheral smear revealed microcytic hypochromic red blood cells with marked anisopoikilocytosis. Target cells, teardrop cells, and pencil cells were observed, along with occasional schistocytes and sickle cells. White blood cell morphology was normal, and platelets were adequate (Table 2 and Figure 1).

**Table 2:** Peripheral smear findings.

Feature	Observation
RBC morphology	Microcytic hypochromic
Anisopoikilocytosis	Present
Target cells	Present
Teardrop cells	Present
Pencil cells	Present
Schistocytes	Few
WBC	Normal morphology
Platelets	Adequate

Biochemical Findings

Total bilirubin was 2.78 mg/dL with indirect bilirubin of 2.29 mg/dL. Lactate dehydrogenase (LDH) was elevated at 655 U/L, and serum haptoglobin was markedly reduced at less than 10 mg/dL. These findings confirmed ongoing hemolysis (Table 3).

**Table 3:** Biochemical profile of the patient. LDH: lactate dehydrogenase; CRP: C-reactive protein; ESR: erythrocyte sedimentation rate

Parameter	Value	Reference Range	Interpretation
Total bilirubin	2.78 mg/dL	0.2-1.2 mg/dL	Elevated
Indirect bilirubin	2.29 mg/dL	<1.0 mg/dL	Elevated
LDH	655 U/L	220-440 U/L	Elevated
CRP	15-16 mg/L	<6 mg/L	Elevated
ESR	Up to 39 mm/hr	<10 mm/hr	Elevated

Enzyme and Iron Studies

Glucose-6-phosphate dehydrogenase (G6PD) test (by the kinetic method) showed a value of 160.90 U/dL of blood, while quantitative G6PD was 20.37 U/g Hb (reference range: 4.62-13.5), which was within normal limits. Iron studies were also within normal ranges. These findings excluded G6PD deficiency and iron deficiency anemia.

Infectious and secondary workup

The anti-malarial antibody test was negative to rule out tropical splenomegaly syndrome. Serological tests for HIV, hepatitis B surface antigen, and hepatitis C antibody were all negative. Blood cultures and urine cultures showed no growth. Secondary causes of hemolysis were effectively excluded (Table 4).

**Table 4:** Evaluation of differential diagnoses in hemolytic anemia. G6PD: glucose-6-phosphate dehydrogenase; LDH: lactate dehydrogenase

Differential Diagnosis	Investigation Performed	Result	Interpretation
Autoimmune hemolytic anemia	Direct Coombs test (DCT)	Negative	Excluded immune-mediated hemolysis
G6PD deficiency	G6PD level assay	Normal	Excluded enzyme deficiency
Malarial hemolysis	Anti-malarial antibody test	Negative	Tropical infectious cause ruled out
Sepsis-related hemolysis	Blood culture	No growth	No evidence of bacteremia
	Urine culture	No growth	No evidence of urinary infection
Viral-induced hemolysis	HIV serology	Negative	Excluded
	Hepatitis B surface antigen	Negative	Excluded
	Hepatitis C antibody	Negative	Excluded
Hemolysis	Serum haptoglobin	<10 mg/dL (Low)	Supports ongoing hemolysis
	LDH	Elevated (655 U/L)	Supports hemolysis
	Indirect bilirubin	Elevated	Supports hemolysis
Iron deficiency anemia	Iron studies	Normal	Excluded

Hemoglobin analysis

**Figure 2:**
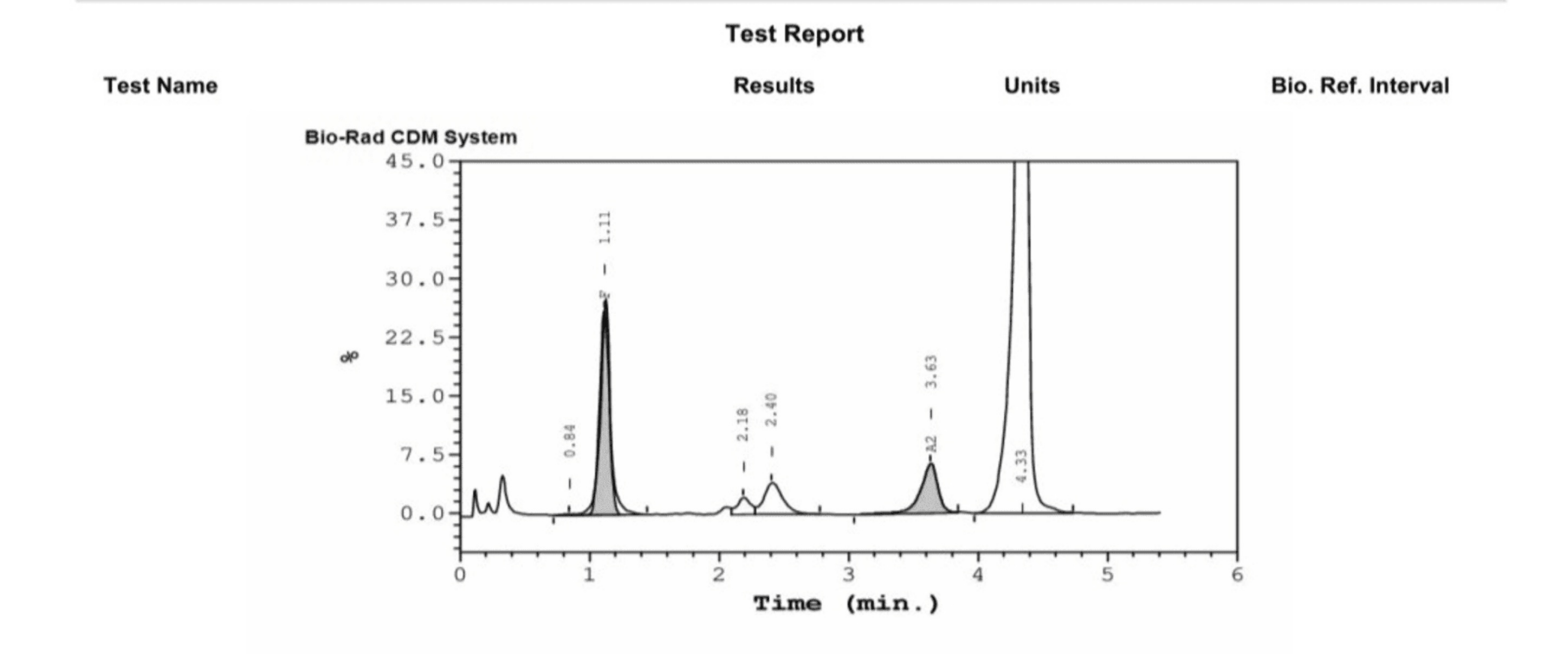
Hemoglobin HPLC chromatogram demonstrating predominant HbS with elevated HbF and HbA2, consistent with compound heterozygous sickle cell-β thalassemia. HPLC: high-performance liquid chromatography; HbS: hemoglobin S; HbF: fetal hemoglobin; HbA2: hemoglobin A2

High-performance liquid chromatography (HPLC) revealed HbS 73.1%, HbF 15%, HbA2 6.3%, and HbA 4.3%. These findings were diagnostic of compound heterozygous sickle cell-beta thalassemia (Table 5 and Figure 2).

**Table 5:** High-performance liquid chromatography (HPLC) hemoglobin analysis. HbS: hemoglobin S; HbF: fetal hemoglobin; HbA2: hemoglobin A2; HbA: hemoglobin A

Hemoglobin Fraction	Value (%)	Reference Range	Interpretation
HbS	73.1	0%	Markedly elevated
HbF	15	<1.5%	Elevated
HbA2	6.3	1.5-3.5%	Elevated
HbA	4.3	83-90%	Reduced

Imaging

**Figure 3:**
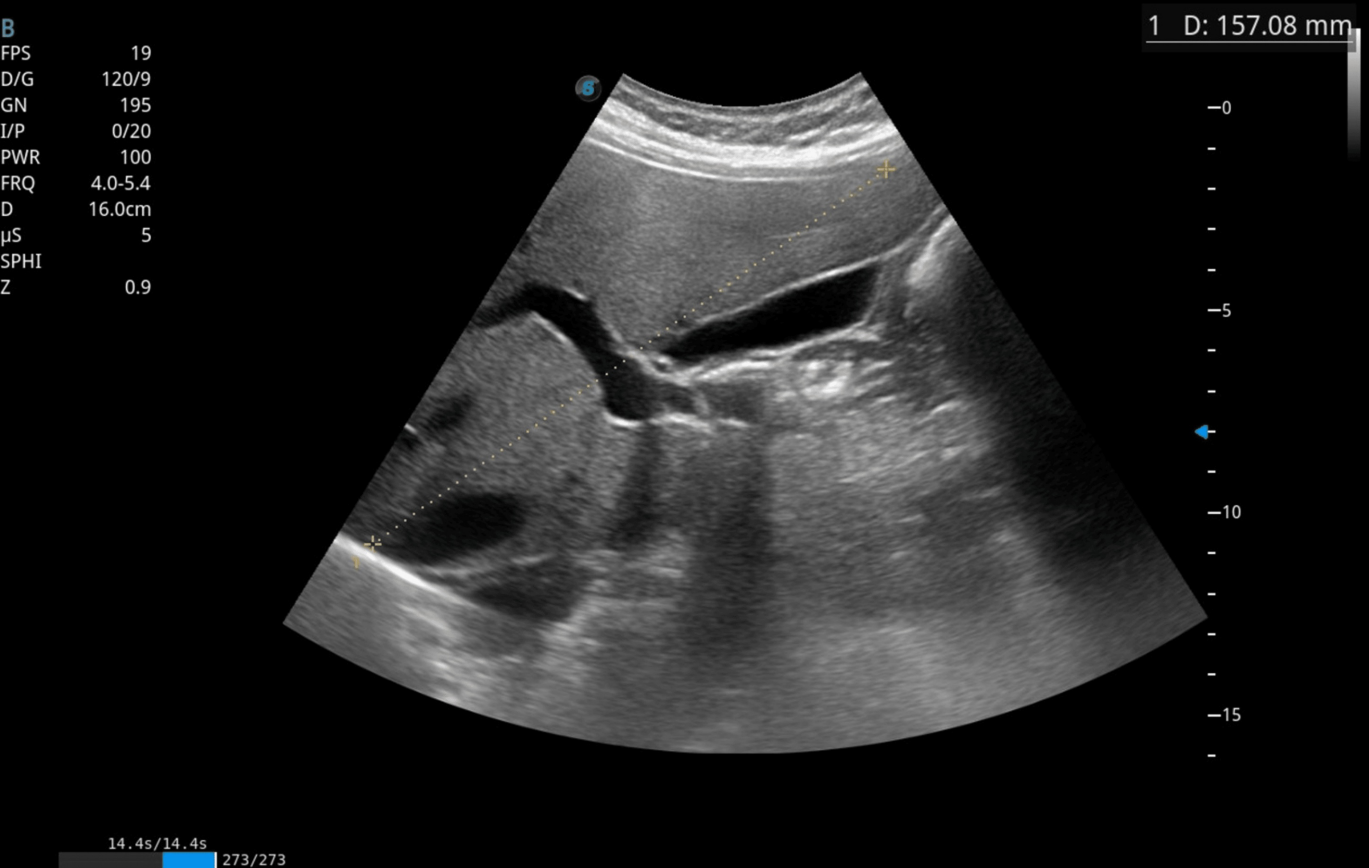
Abdominal ultrasound showing splenomegaly (approximately 15.7 cm) in a patient with compound heterozygous sickle cell-beta thalassemia and chronic hemolytic anemia.

**Figure 4:**
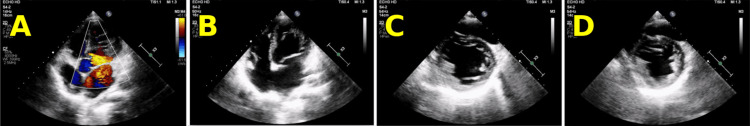
Multiplanar transthoracic echocardiographic views demonstrating prominent left ventricular apical trabeculations in compound heterozygous sickle cell-β thalassemia. (A) Apical four-chamber view with color Doppler demonstrating blood flow within deep intertrabecular recesses at the left ventricular apex. (B) Two-dimensional apical four-chamber view confirming persistent apical trabeculations. (C) Parasternal short-axis view at the apical level demonstrating excessive trabecular meshwork within the left ventricular cavity. (D) Additional parasternal/apical view demonstrating persistence of trabeculations throughout the cardiac cycle.

Ultrasound of the abdomen demonstrated splenomegaly measuring approximately 15.7 cm (Figure 3). Echocardiography revealed mild global hypokinesia, a bicuspid aortic valve, and mild aortic and mitral regurgitation. Prominent left ventricular apical trabeculations were noted, raising consideration of adaptive cardiac remodeling versus non-compaction cardiomyopathy (Figure 4 and Table 6). Cardiac magnetic resonance imaging was not performed; hence, definitive classification of the trabeculation pattern could not be established.

**Table 6:** Imaging and cardiac findings. LV: left ventricular; MR: mitral regurgitation; AR: aortic regurgitation

Investigation	Findings
Ultrasound abdomen	Splenomegaly (~15.7 cm)
Chest X-ray	No significant abnormality
Echocardiography	Mild MR, bicuspid aortic valve, mild AR
LV function	Mild global hypokinesia
LV apex	Prominent trabeculations (adaptive remodeling vs. non-compaction)

Management and follow-up

The patient was initiated on hydroxyurea at a dose of 20 mg/kg/day to reduce hemolysis and improve clinical outcomes, along with folic acid supplementation [6]. Blood transfusion was deferred as the patient was hemodynamically stable without features of symptomatic anemia. The patient was counseled regarding the chronic nature of the disease and advised to undergo regular follow-up. Family screening was recommended to identify carrier status and facilitate genetic counseling. Cardiology follow-up was planned for further evaluation and monitoring of left ventricular trabeculation, with consideration for cardiac MRI to better characterize the trabeculation pattern.

## Discussion

Sequential testing in this patient revealed the diagnosis by ruling out alternative causes one by one. The presence of anemia with reticulocytosis, indirect hyperbilirubinemia, elevated LDH, and markedly reduced serum haptoglobin confirmed active hemolysis. The combination of indirect hyperbilirubinemia (2.29 mg/dL), elevated LDH (655 U/L), and markedly reduced haptoglobin (<10 mg/dL) provided objective biochemical confirmation of ongoing hemolysis.

The negative direct Coombs test excluded immune-mediated hemolysis. The systematic exclusion approach documented in our differential workup table demonstrates how we ruled out infectious causes (negative malaria antibodies, blood cultures), immune causes (negative Coombs), and enzymatic defects (normal G6PD), narrowing the differential toward intrinsic red cell defects. Normal iron studies excluded the common pitfall of misdiagnosing microcytic anemia as iron deficiency. The severe microcytosis (MCV 65-70 fL) combined with elevated RDW (28.1%) and reticulocytosis (5%) was the critical clue that distinguished this from simple iron deficiency anemia, which typically shows low reticulocyte counts. This pointed us toward an intrinsic red cell problem.

Finding microcytosis alongside hemolysis was the critical clue. Delayed diagnosis of sickle cell disease carries significant morbidity risk, making early recognition essential [1]. Homozygous sickle cell disease usually shows normocytic red cells, but this patient had small cells and high HbA2, both suggesting co-inherited thalassemia [3,7]. Hemoglobin HPLC analysis definitively established the diagnosis, showing the characteristic pattern of HbS dominance (73.1%), elevated HbF (15%), elevated HbA2 (6.3%), and crucially, the presence of residual HbA (4.3%), which classified this as a β+ phenotype rather than β0. Getting this distinction right matters for proper management.

Epidemiological significance

This case is epidemiologically relevant given the patient's origin from Assam, a state in Northeast India with a distinctive hemoglobinopathy profile. HbS-β thalassemia is reported to be rare in India and particularly uncommon in Assam, where HbE-related hemoglobinopathies predominate [4,5] in different ethnic groups [8,9]. The occurrence of HbS-β thalassemia in this patient from Assam, where HbE variants dominate, shows why we should do complete hemoglobin testing even in areas where specific variants are uncommon.

Chronic anemia causes cardiac remodeling - the heart adapts to the sustained high-output state. This patient's prominent trabeculations most likely reflect that adaptation, not a primary heart muscle disease. The echocardiographic demonstration of prominent left ventricular apical trabeculations in multiple cardiac views, combined with the patient's chronic severe anemia, supports the interpretation of adaptive cardiac remodeling rather than primary cardiomyopathy. Current evidence indicates that chronic hemoglobinopathies are associated with increased left ventricular trabeculation due to increased preload and high cardiac output states, and these adaptive changes typically do not cause adverse clinical effects [10,11].

While definitive classification would require cardiac MRI with specific non-compaction criteria [12,13], this finding shows how chronic, untreated anemia affects the heart. Whether early hydroxyurea can prevent these cardiac changes needs more research [14]. Cardiac MRI offers the clearest picture here - techniques like late gadolinium enhancement and T2* mapping help separate adaptive changes from true heart muscle disease in patients with chronic hemolytic conditions. In beta-thalassemia and similar disorders, heart problems cause considerable mortality, so routine cardiac checks and follow-up imaging are crucial [15,16].

Starting hydroxyurea at 20 mg/kg/day follows current evidence-based recommendations. Hydroxyurea increases HbF levels and improves hemolytic parameters, which is associated with significant clinical benefits, including reduction in vaso-occlusive episodes [6,14].

## Conclusions

This case shows why systematic workup matters when evaluating hemolytic anemia with unusual red cell features. Microcytic hemolytic anemia should raise suspicion for compound hemoglobinopathies - not just iron deficiency or simple thalassemia trait. Hemoglobin analysis is essential for accurate diagnosis, and early identification enables appropriate management and genetic counseling. The epidemiological rarity of HbS-β thalassemia in Assam, where HbE variants predominate, reinforces the need for comprehensive hemoglobin analysis regardless of regional prevalence patterns.

When we see cardiac changes in chronic anemia, we need to consider the hemodynamic picture and plan cardiac follow-up, potentially including advanced imaging. Early initiation of hydroxyurea in sickle cell-beta thalassemia can reduce hemolysis and prevent long-term complications. This case illustrates how sequential diagnostic exclusion can reveal uncommon hemoglobinopathies, particularly in regions where certain variants are rare despite high overall prevalence of related disorders.
